# Post-class naps boost declarative learning in a naturalistic school setting

**DOI:** 10.1038/s41539-018-0031-z

**Published:** 2018-08-21

**Authors:** Thiago Cabral, Natália B. Mota, Lucia Fraga, Mauro Copelli, Mark A. McDaniel, Sidarta Ribeiro

**Affiliations:** 1 Laboratory of Memory, Sleep and Dreams, Brain Institute, Federal University of Rio Grande do Norte (UFRN), Natal, Brazil; 2 Department of Physics, Federal University of Pernambuco (UFPE), Recife, Brazil; 3 State School Berilo Wanderley, Natal, Brazil; 4 Department of Psychological and Brain Sciences, Washington University, St. Louis, USA

## Abstract

Laboratory evidence of a positive effect of sleep on declarative memory consolidation suggests that naps can be used to boost school learning in a scalable, low-cost manner. The few direct investigations of this hypothesis have so far upheld it, but departed from the naturalistic setting by testing non-curricular contents presented by experimenters instead of teachers. Furthermore, nap and non-nap groups were composed of different children. Here we assessed the effect of post-class naps on the retention of Science and History curricular contents presented by the regular class teacher to 24 students from 5th grade. Retention was repeatedly measured 3–4 days after content learning, with weekly group randomization over 6 consecutive weeks. Contents followed by long naps (>30 min), but not short naps (<30 min), were significantly more retained than contents followed by waking (Cohen’s *d* = 0.7962). The results support the use of post-class morning naps to enhance formal education.

## Introduction

Sleep benefits learning and shows potential to improve education worldwide.^1–3^ Delaying the beginning of classes to increase the amount of pre-learning sleep improves attendance and decreases depression.^4–6^ Laboratory studies show that post-learning sleep improves declarative memory consolidation in adults^7–9^ as well as children.^10–12^ Yet, few studies of post-learning naps have been performed inside schools:^13,14^ in pre-schoolers, mid-day naps benefit spatial recall 24 h after learning;^13^ in adolescents, post-learning naps improve retention after 5 days.^14^

Albeit promising, these school-based studies have major caveats: learning was mediated by experimenters instead of teachers, tests comprised non-curricular contents, and nap/non-nap comparisons were made in groups composed of different children. We set out to investigate post-learning naps in a more naturalistic school context, by testing curricular contents presented by the regular teacher in a cross-over design with weekly randomization that allowed each student to participate in all groups.

## Results

**Fig. 1:**
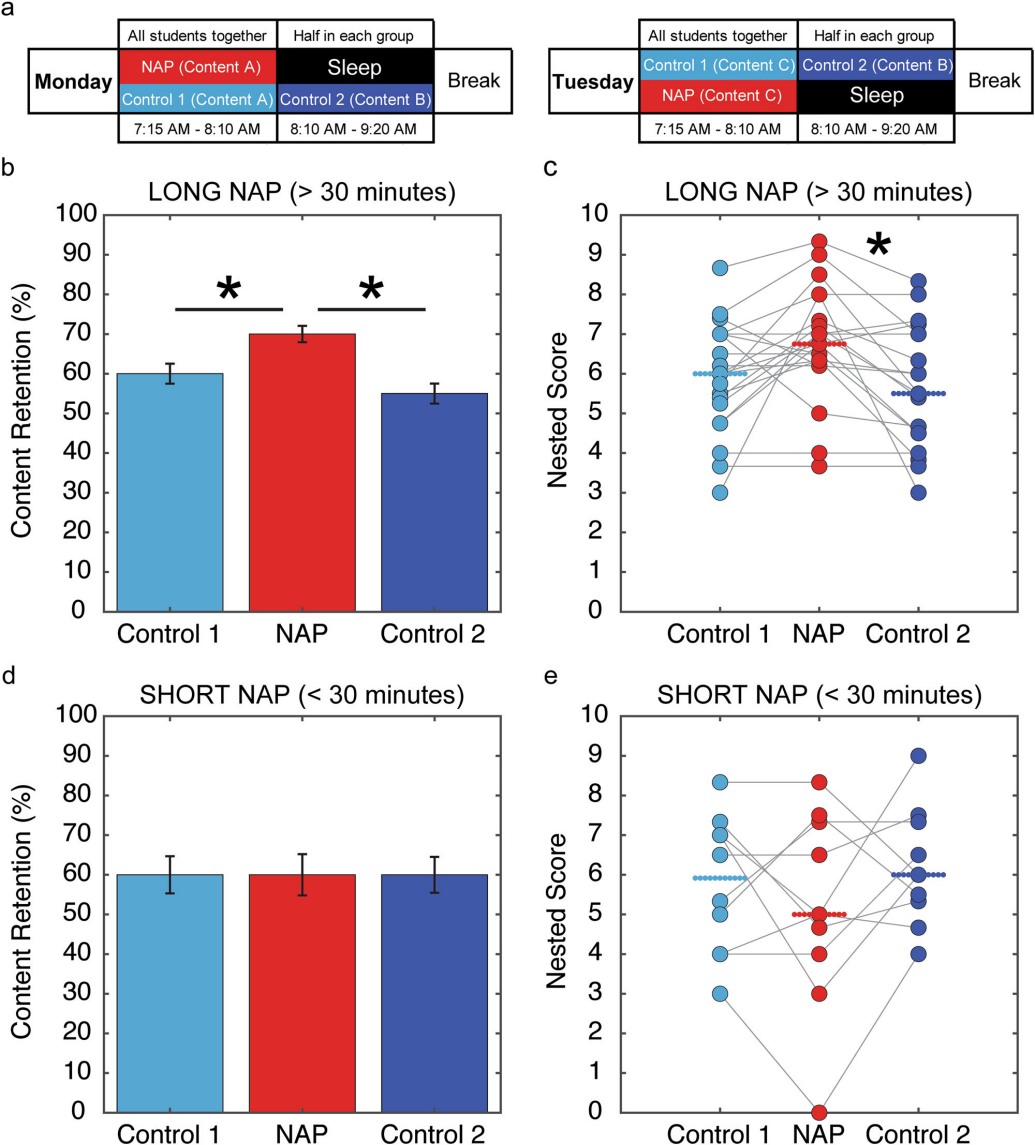
Experimental design and content retention for Science and History curricular contents presented for 6 consecutive weeks of sleep intervention. **a** On Monday, 24 students attended the same class on content A, followed by sleep time for 12 students randomly chosen each week, while the other 12 students attended class on content B. On Tuesday, all students attended the same class on content C, followed by sleep time for the students who on Monday did not have it, while the other half attended class on content B, which they had missed on Monday. Classes followed by sleep time were called NAP (contents A or C); classes followed by another content from the same discipline were called Control 1 (control with specific interference; contents A or C); classes followed by a break were called Control 2 (control with non-specific interference; always content B). **b** In average, content retention was significantly higher for contents followed by long naps (>30 min) than for contents followed by waking activities with non-specific or specific interference. **c** A nested analysis of individual performances showed significant gains for long naps, in comparison with non-specific waking interference. Short naps did not show benefits, neither for **d** group averages nor **e** individual nested data. Bars represent the median performance for each condition, with error bars representing standard error and each individual mean performance represented by dots. Statistical significant difference between groups using Wilcoxon Ranksum test corrected for 2 comparisons is represented by *

Contents followed by long naps (>30 min) were significantly better retained than contents followed by a different lecture on the same discipline (Control 1), when assessed by trial averages (*p* = 0.0246/Cohen’s *d* = 0.4119, Fig. 1b), with a marginal trend when the data were nested per subject (*p* = 0.0435/Cohen’s *d* = 0.6225, Fig. 1c). Contents followed by long naps were also better retained than contents followed by a break (Control 2) when assessed either by trial averages (*p* = 0.0013/Cohen’s *d* = 0.5344, Fig. 1b) or nested per subject (*p* = 0.01725/Cohen’s *d* = 0.7962, Fig. 1c). No significant differences were observed when short naps (<30 min) were considered (Fig. 1d, e). Statistics are summarized in Table 1.

**Table 1:** Statistical results comparing performances of students on the contents followed by sleep (NAP) × waking controls with specific (Control 1) or non-specific (Control 2) interference

**Trials** **>30**′ **(72)**	**All Trials Analysis**	**Subjects** **>30**′ **(21)**	**Nested Analysis**
KS	0.0000	KS	0.0000
Levene	0.1385	Levene	0.9231
KW	**0.0047**	KW	**0.0312**
WR Control 1 × NAP	**0.0246**	WR Control 1 × NAP	0.0435
WR Control 2 × NAP	**0.0013**	WR Control 2 × NAP	**0.0172**

**Trials** **<30**′ **(21)**	**All Trials Analysis**	**Subjects** **<30**′ **(10)**	**Nested Analysis**
KS	0.0000	KS	0.0000
Levene	0.7378	Levene	0.2742
KW	0.9197	KW	0.6296
WR Control 1 × NAP	0.8988	WR Control 1 × NAP	0.7325
WR Control 2 × NAP	0.7124	WR Control 2 × NAP	0.3631

The table shows *p* values from Kolmogorov–Smirnoff test (KS) and the Levene test. Since the data were not normally distributed, we used the Kruskal–Wallis test (KW) followed by the Wilcoxon Ranksum test for pairwise comparisons (Bonferroni correction for 2 comparisons, *α* = 0.025). Boldface indicates statistically significant differences. Two analyses were performed: (i) considering all trials (“All Trials Analysis”), and (ii) using the average of all results from the same subject in each condition (“Nested Analysis”). Number of trials/subjects are indicated in parenthesis

There was no significant performance difference when contents where presented 3 or 4 days before the test, neither for contents A/C followed by nap, nor for contents A/C followed by another class, nor for content B followed by the break (Supplementary Table S1). There was a significant difference between Science and History considering all groups due to a difference in the Control 2 group (Supplementary Table S2).

## Discussion

Naps lasting 30–60 min seem to boost memory retention of curricular contents by ~10%, similar to our previous assessment in students of a similar age range.^14^ Also, the lack of effect for shorter naps agrees with prior laboratory findings.^9^ Napping was designed to begin at 08:15, after the first morning class. Though there is typically low sleep pressure at this time,^15^ students did not have problems falling asleep. Sun dawn in Natal occurs around 05:00, students wake up around 5:30, and they often come drowsy to school due to sleep debt.^16^ Morning naps show low slow-wave sleep,^15^ and are comprised mostly of Stage 2 sleep and rapid-eye-movement (REM) sleep.^17^ REM-rich naps benefit creativity,^18^ whereas Stage 2 sleep has been related to declarative memory.^19^ In our experiment, the material learned corresponded to declarative contents, and the testing involved rote learning and the association of multiple contents, but not abstract or creative thinking. Therefore, the morning nap benefits were likely related to Stage 2 sleep, not REM sleep.

The nested analysis consisted of averaging the scores per subject/condition, thus avoiding mixing the variance within-subject with the variance across subjects. Overall this multilevel approach (Fig. 1c) supported the conclusion drawn from the all-trials approach (Fig. 1b). Note that the latter does not show the individual differences detected by the nested analysis. The equivalent performances for contents presented 3 or 4 nights before the test do not rule out the possibility that only the combination of long naps with a full night of sleep can lead to learning gains, because a single night may have sufficed.^20–22^

One important caveat is that we did not attempt to monitor rest/activity (e.g., actigraphy, electroencephalography) because of the long duration of the study (6 continuous weeks), and the possible unintended effects of using recording devices in a low socioeconomic status (SES) community. We opted not to record activity because there was a big chance that it would not be properly sampled, and would generate emotional and cognitive confounds impossible to control. Another caveat worth noting is that the lack of assessment of sleep inertia at awakening. This occurred because the school schedule was quite squeezed by the need to end the daily school activities at 12:00.

The design features of our naturalistic experiment were motivated by evidence that school learning is improved by novelty.^23^ In our prior study of post-learning sleep with low SES Brazilian students,^14^ we observed that the presence of a graduate student performing the study caused quite a stir among the students. Teachers also remarked that the students paid extra attention to the non-curricular contents. This means that the positive effects on learning attributed to sleep could possibly derive from an interaction between novelty and sleep. The current experiment was designed to bypass these confounds by moving much closer to the real-life school situation, with the aim of achieving a viable, scalable improvement in school learning. It was also designed to probe whether post-learning sleep could make efficient use of morning classes, which are negatively impacted by overnight (pre-learning) sleep debt,^16^ a disruptor of declarative learning.^24^

Because both Control groups were subjected to interference after the target instruction, the positive influence attributed to sleep could in principle derive from lack of interference in the nap condition. However, contents followed by short naps did not show retention gains. This suggests that the benefits attributed to post-learning naps are not artefacts of reduced interference. Rather, the results obtained with our ecological approach point to the usefulness of relatively long post-learning morning naps to enhance school learning. Future studies shall determine whether the long-term benefits of post-learning naps may grow like compound interest.

## Methods

### Subjects

A sample of 24 students (5th grade, 12 female and 12 male, age 10.83 ± 0.24 years, mean ± s.e.m.) was studied. An independent comparable sample was studied to establish equivalency among content parts (*N* = 26). Consent forms were signed by parents or legal guardians, and the study was approved by the UFRN Research and Ethics Committee (#461.394). The research complied with all relevant ethical regulations.

### Experimental design

Data collection took place entirely at school. The experiment lasted 6 weeks, with Science contents during the first 3 weeks (The Human Body, Health & Locomotion, Nervous System), and History contents during the last 3 weeks (Slavery, Immigration, Slave Rebellions). For every week, the standard curricular contents were separated in three equivalent parts (A, B, and C), and presented by the regular class teacher (L.F.). To ensure content equivalency, first we asked L.F. which contents she would normally cover during that period. Based on this information and on the teaching materials normally employed by her, we separated the weekly contents into three parts, and created 10 exam questions per part. Parts A, B, and C were then tested on an independent sample (*N* = 26) to ascertain equivalency. No significant difference across parts was detected (Supplementary Table S3). On Monday mornings, all students were exposed to a 60 min lecture on content A (Fig. 1a). After the lecture from 07:15 to 08:15, they were randomly sorted into sleep and non-sleep sets of equal size. From 8:15 to 9:20, half of the students were conducted to a quiet dark room with mats, received eye masks, and were invited to sleep for up to 60 min, while the other half remained in class to receive a lecture on content B (Fig. 1a). After spontaneously waking up, students in the sleep set were asked to estimate how long they have slept, and this self-report was compared to the teacher’s observations to determine whether the naps were long (>30 min) or short (<30 min). At 9:20, all students returned to their ordinary schedule, with a 20 min break followed by more classes until 12:00. On Tuesday mornings, the same procedures were performed for contents B and C: All students attended together a lecture on content C from 07:15 to 08:15, then students sorted into the sleep set on the previous day received a lecture on content B from 8:15 to 9:20, while the other students were invited to sleep (Fig. 1a). On Fridays at 7:15, retention of content parts A, B, and C was assessed by multiple-choice tests with 10 questions per part. Groups were labeled according to whether they were followed by sleep (NAP), followed by another lecture with different content within the same topic (control with specific interference, Control 1), or class break (control with non-specific interference, Control 2). Lecture duration (50 min) matched Brazilian standards. Students slept up to 60 min, a duration that benefits declarative memory consolidation in the laboratory^7–12^ and can fit most school programs. The nap administration time (8:15) was directly related to the start time of classes (07:15), which corresponds to the standard start time for most Brazilian schools. Though this start time is on the early end of the spectrum, it is not rare across the world, including the USA and China (Supplementary Table S4).

### Statistical analyses

There was no imputation of missing data, i.e., students who missed the classes and/or the test were not included in the weekly sample. Data were not normally distributed (Table 1). Accordingly, the Kruskal–Wallis test was conducted, followed by two-sided Wilcoxon Ranksum tests for pairwise comparisons (NAP × Control 1 or NAP × Control 2), with Bonferroni correction. The analysis considered all trials from all subjects in each condition (“All Trials” analysis), as well as averages of all trials per condition per subject (“Nested” analysis). All the statistical analyses performed with MATLAB 2017 software.

### Data availability

All the raw data are shown in Supplementary Table S5.

## Electronic supplementary material

Supplementary Tables

## Electronic supplementary material

**Supplementary information** accompanies the paper on the *npj Science of Learning* website (10.1038/s41539-018-0031-z).
