# Supplementary material for: Post-class naps boost declarative learning in a naturalistic school setting
Source: NPJ Sci Learn. 2018 Aug 21;3:14. doi: 10.1038/s41539-018-0031-z (PMC6220199; doi:10.1038/s41539-018-0031-z)
Supplement: Supplementary file 1 — Supplementary Tables [file 41539_2018_31_MOESM1_ESM.docx]

**Supplementary Tables**

**Suppl. Table S1:** Comparison of contents presented 3 or 4 days before the test Page 2

**Suppl. Table S2:** Science versus History comparison Page 3

**Suppl. Table S3:** Content comparison using the independent sample Page 4

**Suppl. Table S4:** Earliest school start times across countries Page 5

**Suppl. Table S5:** Raw data Pages 6,7

**Supplementary Table S1: Comparison of contents presented 3 or 4 days before the test.**

| **Monday x Tuesday** | **Monday** | | | **Tuesday** | | | **Corrected α = 0.0167** |
| --- | --- | --- | --- | --- | --- | --- | --- |
| **Quantiles** | **25%** | **Median** | **75%** | **25%** | **Median** | **75%** | **Ranksum p value** |
| **NAP (Content A x Content C)** | 6.00 | 7.00 | 8.00 | 5.00 | 6.00 | 8.00 | 0.1040 |
| **Control 1 (Content A x Content C)** | 4.00 | 6.00 | 7.00 | 5.00 | 6.00 | 8.00 | 0.2395 |
| **Control 2 (Content B)** | 4.00 | 5.00 | 7.00 | 5.00 | 6.00 | 8.00 | 0.1343 |

**Supplementary Table S2: Science versus History comparison.**

| **Science x History** | **Science** | | | **History** | | | **Corrected α = 0.0167** |
| --- | --- | --- | --- | --- | --- | --- | --- |
| **Quantiles** | **25%** | **Median** | **75%** | **25%** | **Median** | **75%** | **Ranksum p value** |
| **All** | 4.00 | 6.00 | 7.00 | 5.00 | 6.50 | 8.00 | **0.0139** |
| **NAP** | 5.00 | 6.00 | 8.00 | 6.00 | 7.00 | 8.00 | 0.1404 |
| **Control 1** | 5.00 | 6.00 | 7.00 | 4.00 | 6.00 | 8.00 | 1.0000 |
| **Control 2** | 3.75 | 5.00 | 6.25 | 5.00 | 6.00 | 8.00 | **0.0047** |

**Supplementary Table S3: Content comparison using the independent sample.**

| **Kruskal-Wallis** | **p value** | 0.9509 |
| --- | --- | --- |
| **Ranksum AxB** | **p value** | 0.7456 |
| **Ranksum AxC** | **p value** | 0.8285 |
| **Ranksum BxC** | **p value** | 0.9822 |

**Supplementary Table S4: Earliest school start-times across countries.**

| **Country** | **Earliest Start Time** | **References** |
| --- | --- | --- |
| Brazil | 07:00 | Silva et al. (2005) Arq Neuropsiquiatr. 63(2B):402-6; https://elearninginfographics.com/school-days-around-world-infographic/ |
| China | 07:00 | Li et al. (2013) PLoS One 8(7):e67928; Chenet al. (2014) Sleep Med. 15(11):1345-53; https://elearninginfographics.com/school-days-around-world-infographic/ |
| USA | 07:00 | Carskadon et al. (1998) Sleep 21(8):871-81; Wolfson (2007) Behav Sleep Med. 5(3):194–209; Carrell et al. (2011) Am Econ J: Econ Policy. 3(3):62–81; Ming et al.(2011) Clin Med Insights Circ Respir Pulm Med. 5:71–79; Paksarian et al. (2015) Am J Public Health. 105(7):1351–1357; https://nces.ed.gov/pubs2017/2017071.pdf |
| Turkey | 07:00 | Yilmaz et al. (2011) Turk J Pediatr. 53(4):430–436 |
| Croatia | 07:00 | Milić et al. (2014) Coll Antropol.38(3):889–894 |
| Israel | 07:10 | Epstein et al. (1998) Sleep 21(3):250–256; Lufi et al. (2011) J Clin Sleep Med. 7(2):137–143 |
| Singapore | 07:30 | Lo et al (2018) Sleep, zsy052, https://doi.org/10.1093/sleep/zsy052 |
| Switzerland | 07:30 | Strauch & Meier (1998) Sleep 11(4):378-86; https://elearninginfographics.com/school-days-around-world-infographic/ |
| Australia | 07:30 | Short et al. (2013) Health Educ Behav. 40(3):323–330; http://www.education.vic.gov.au/school/principals/spag/management/Pages/hours.aspx |
| Hong Kong | 07:35 | Zhang et al. (2010) J Pediatr. 156(4):606–612. e605 |
| Spain | 08:00 | Escribano et al. (2014) Chronobiol Int. 31(6):761–769 |
| Canada | 08:30 | Gariépy et al. (2017), School start time and sleep in Canadian adolescents. J Sleep Res, 26: 195-201 |
| Norway | 08:30 | Vedaa et al. (2012) Scand J Educ Res. 56(1):55–67 |
| New Zealand | 09:00 | Borlase et al. (2013) Sleep Biol Rhythms 11(1):46–54 |

**Supplementary Table S5: Raw data.** NAP, Control 1 and Control 2 data represent tests scores ranging from 0-10. Nap duration data represent estimated minutes of sleep.

| **Subject #** | **Week 1** | | | | **Week 2** | | | | **Week 3** | | | |
| --- | --- | --- | --- | --- | --- | --- | --- | --- | --- | --- | --- | --- |
|  | **NAP** | **CTR1** | **CTR2** | **Nap duration** | **NAP** | **CTR1** | **CTR2** | **Nap duration** | **NAP** | **CTR1** | **CTR2** | **Nap duration** |
| 1 |  |  |  |  | 6 | 8 | 7 | >30 | 7 | 5 | 2 | >30 |
| 2 | 6 | 6 | 5 | >30 | 7 | 6 | 6 | >30 | 6 | 8 | 7 | >30 |
| 3 | 7 | 5 | 7 | >30 |  |  |  |  | 6 | 8 | 8 | >30 |
| 4 | 3 | 6 | 4 | >30 |  |  |  |  | 4 | 6 | 4 | >30 |
| 5 |  |  |  |  | 8 | 7 | 4 | >30 | 5 | 7 | 5 | >30 |
| 6 | 4 | 2 | 5 | <30 | 5 | 5 | 4 | <30 | 6 | 5 | 5 | <30 |
| 7 |  |  |  |  |  |  |  |  |  |  |  |  |
| 8 |  |  |  |  | 7 | 5 | 3 | >30 |  |  |  |  |
| 9 |  |  |  |  | 5 | 9 | 6 | >30 | 6 | 8 | 3 | >30 |
| 10 |  |  |  |  |  |  |  |  |  |  |  |  |
| 11 | 6 | 3 | 2 | >30 | 6 | 5 | 3 | >30 | 8 | 7 | 2 | >30 |
| 12 | 8 | 8 | 9 | >30 | 6 | 5 | 3 | >30 |  |  |  |  |
| 13 | 9 | 9 | 10 | >30 | 10 | 8 | 7 | >30 |  |  |  |  |
| 14 | 4 | 6 | 4 | >30 | 10 | 5 | 4 | >30 |  |  |  |  |
| 15 | 2 | 6 | 5 | <30 | 5 | 6 | 8 | >30 | 5 | 7 | 3 | <30 |
| 16 | 8 | 8 | 6 | <30 | 10 | 7 | 6 | >30 | 8 | 7 | 4 | <30 |
| 17 | 0 | 3 | 4 | <30 |  |  |  |  |  |  |  |  |
| 18 |  |  |  |  |  |  |  |  |  |  |  |  |
| 19 | 3 | 7 | 6 | <30 |  |  |  |  | 8 | 3 | 5 | >30 |
| 20 | 4 | 2 | 5 | >30 |  |  |  |  |  |  |  |  |
| 21 | 6 | 7 | 8 | <30 | 8 | 4 | 6 | >30 | 9 | 5 | 3 | >30 |
| 22 |  |  |  |  |  |  |  |  |  |  |  |  |
| 23 | 6 | 5 | 9 | <30 |  |  |  |  |  |  |  |  |
| 24 |  |  |  |  |  |  |  |  | 7 | 5 | 3 | <30 |

| **Subject #** | **Week 4** | | | | **Week 5** | | | | **Week 6** | | | |
| --- | --- | --- | --- | --- | --- | --- | --- | --- | --- | --- | --- | --- |
|  | **NAP** | **CTR1** | **CTR2** | **Nap duration** | **NAP** | **CTR1** | **CTR2** | **Nap duration** | **NAP** | **CTR1** | **CTR2** | **Nap duration** |
| 1 |  |  |  |  |  |  |  |  |  |  |  |  |
| 2 | 5 | 7 | 9 | <30 | 6 | 4 | 5 | >30 | 6 | 7 | 7 | >30 |
| 3 | 7 | 10 | 10 | >30 |  |  |  |  | 7 | 1 | 4 | >30 |
| 4 | 8 | 9 | 6 | >30 |  |  |  |  |  |  |  |  |
| 5 |  |  |  |  |  |  |  |  |  |  |  |  |
| 6 | 7 | 9 | 7 | >30 | 6 | 3 | 5 | >30 | 6 | 6 | 6 | >30 |
| 7 | 8 | 3 | 3 | >30 |  |  |  |  |  |  |  |  |
| 8 | 8 | 7 | 8 | >30 | 7 | 5 | 4 | >30 | 6 | 2 | 7 | >30 |
| 9 | 8 | 10 | 10 | >30 | 6 | 6 | 6 | >30 | 6 | 4 | 5 | >30 |
| 10 | 6 | 9 | 9 | >30 | 9 | 2 | 5 | >30 | 7 | 7 | 5 | >30 |
| 11 | 6 | 8 | 7 | >30 | 7 | 5 | 4 | >30 | 5 | 5 | 5 | >30 |
| 12 | 9 | 4 | 6 | >30 | 7 | 4 | 5 | >30 | 6 | 6 | 4 | >30 |
| 13 |  |  |  |  |  |  |  |  | 9 | 9 | 8 | >30 |
| 14 |  |  |  |  | 7 | 4 | 6 | >30 | 6 | 4 | 4 | >30 |
| 15 | 8 | 7 | 9 | >30 | 8 | 8 | 5 | >30 | 7 | 9 | 8 | <30 |
| 16 |  |  |  |  | 8 | 8 | 8 | >30 | 9 | 10 | 8 | <30 |
| 17 | 5 | 3 | 5 | >30 | 1 | 5 | 2 | >30 | 5 | 3 | 4 | >30 |
| 18 | 4 | 3 | 3 | >30 |  |  |  |  | 4 | 5 | 5 | >30 |
| 19 | 10 | 9 | 10 | >30 | 6 | 9 | 9 | >30 | 8 | 7 | 8 | >30 |
| 20 | 10 | 7 | 9 | >30 | 7 | 6 | 7 | >30 | 7 | 6 | 7 | >30 |
| 21 | 9 | 7 | 6 | >30 | 7 | 6 | 7 | <30 | 8 | 7 | 7 | >30 |
| 22 | 4 | 6 | 5 | <30 |  |  |  |  | 4 | 2 | 8 | <30 |
| 23 |  |  |  |  | 6 | 3 | 4 | <30 | 10 | 8 | 9 | <30 |
| 24 | 8 | 5 | 8 | <30 |  |  |  |  |  |  |  |  |
